# Regulation of Gene Expression by Telomere Position Effect

**DOI:** 10.3390/ijms222312807

**Published:** 2021-11-26

**Authors:** Kyung-Ha Lee, Do-Yeon Kim, Wanil Kim

**Affiliations:** 1 Division of Cosmetic Science and Technology, Daegu Haany University, Gyeongsan 38610, Korea; kyungha.lee@dhu.ac.kr; 2 Department of Pharmacology, School of Dentistry, Kyungpook National University, Daegu 41940, Korea; 3 Department of Biochemistry, Department of Convergence Medical Science, Institute of Health Sciences, School of Medicine, Gyeongsang National University, Jinju 52727, Korea

**Keywords:** telomere, aging, gene expression, telomere position effect, telomere looping

## Abstract

Many diseases that involve malignant tumors in the elderly affect the quality of human life; therefore, the relationship between aging and pathogenesis in geriatric diseases must be under-stood to develop appropriate treatments for these diseases. Recent reports have shown that epigenetic regulation caused by changes in the local chromatin structure plays an essential role in aging. This review provides an overview of the roles of telomere shortening on genomic structural changes during an age-dependent shift in gene expression. Telomere shortening is one of the most prominent events that is involved in cellular aging and it affects global gene expression through genome rearrangement. This review provides novel insights into the roles of telomere shortening in disease-affected cells during pathogenesis and suggests novel therapeutic approaches.

## 1. Introduction

Telomeres are repetitive sequences that are located at the ends of human chromosomes and are known to constitute ribonucleocomplexes that play a vital role in genome stability. One of the most prominent events that can occur due to the imperfect telomere structure would be an end-to-end fusion between telomeres. Telomere fusion is closely related to the early development of cancer cells, and it is known to induce aneuploidy in cancer cells through the rearrangement of chromosomes [1,2,3]. It has been shown that TRF2 (Telomeric repeat binding factor 2) and RAP1 (Repressor/activator protein 1) proteins play central roles in the inhibition of HR (Homology-directed repair) at chromosomal ends, followed by telomere fusion [4,5,6,7]. Since TRF2 and RAP1 are major factors in the human shelterin complex [8], maintaining an appropriate and sufficient telomere length is very important for genome stability.

## 2. Roles of Telomeres

Telomeres are sequences that exist at the end of chromosomes and are composed of TTAGGG repeats in vertebrates [9]. The length of telomeres decreases linearly in proportion to the number of cell divisions. Human fibroblasts have been a traditional cellular model for aging studies, and it is well known that the length of the telomeres determines the replicative capacity of fibroblasts [10]. Thus, this phenomenon could be used as a reliable marker of aging. In particular, the methodology of measuring lymphocyte telomeres in the blood is commonly used in longitudinal and cross-sectional studies of aging by showing a linear decrease over time [11]. In general, the average initial telomere length in vivo was around 10 kb, and it decreases by half after the age of 80 in humans [12]. Although many studies on decreases in telomere length by aging have been conducted in peripheral blood mononuclear cells (PBMCs), a study has also revealed that the decrease in telomere length is distinct in T-cells, B-cells, and monocytes [13]. The length of telomeres decreases by 23 to 47 base pairs per year [11] and the attrition rate varies depending on individual genetics such as the length of the telomeres at birth [14]. However, most scientists in this field assume that the age-related decline in telomere length over time is obvious.

The primary function of telomeres is to stabilize chromosome integrity. Incomplete DNA replication in human cells causes a constant loss of terminal telomeric repeats. Thus, the existence of telomeres at the very end of the human genome allows for them to serve as a protective nucleoprotein structure that prevents the loss of information of sub-telomeric genes [15]. Telomeres form specific T-loop and D-loop structures to prevent them from being recognized as DNA double-strand breaks, which is a representative signal of DNA damage [16,17,18]. In a recent study, phosphorylation at Ser365 of TRF2 by CDK2 (Cyclin-dependent kinase 2) was suggested as a major regulation controlling the structural dynamics of the T-loop during the cell cycle [19]. Regulation of T-loop dynamics via this phospho-switch that is present in TRF2 will affect the blocking of unwanted DNA damage response during the cell cycle. The T-loop has also been suggested to contribute to stabilizing the length and structure of telomeres since telomeres fold back into loops and self-prime their replicative extension [20]. The authors further hypothesized that the mechanism that is present in the self-primed telomere extension from the T-loop could likely be associated with the expansion of disease-related nucleotide repeats in Huntington’s disease, fragile X syndrome, and amyotrophic lateral sclerosis.

The unique structure at the end of the telomere is assisted by internal complementary interactions between repeats and trans-acting proteins such as TRF1, TRF2, and POT1 [21,22]. These proteins are part of the human shelterin complex, which consists of proteins that contribute to the structural stabilization and maintenance of telomeres by protecting them from DNA repair machineries such as ATM (ataxia-telangiectasia mutated) and ATR (ATM- and Rad3-related) [23,24,25]. Another function of the shelterin complex is to regulate telomerase activity [26,27]. Therefore, proper maintenance of telomere structure is vital for the stability of the human genome.

The continuous loss of telomeres induces M1 senescence, which causes cell division arrest, followed by an M2 crisis that involves further extensive telomere attrition in the absence of cell cycle checkpoints [28,29]. Therefore, telomeres that are shorter than a certain length are strongly associated with replicative senescence, and the length of telomeres represents a clock for aging in human cells, which is referred to as ‘replicometers’ [30]. Replicative senescence is indicative of cells that have undergone enough cell divisions to be considered as having accumulated sufficient DNA mutations to transform into cancer cells. Aging can be caused not only by the loss of telomeres, but also by oncogene expression or DNA damage, all of which are related to genomic instability [31,32]. Therefore, induced senescence due to telomere attrition stops the division of cells at an appropriate time and sufficiently reduces the probability of an event that creates cancer cells by chance; thus, it paradoxically plays a role in ensuring the prolonged survival of the individual.

Telomeres are essential cellular components that must be maintained for the continuous division of cells. Therefore, stem cells prevent the loss of telomeres by expressing telomerase reverse transcriptase, which induces elongation of the telomeres [33]. Telomerase is highly expressed in the fetus and performs essential functions in human development during extensive cell division in the fetal stage [34]. After completion of the fetal stage, telomerase is not expressed in somatic cells, except for specific stem cells, and therefore most of the proliferative human cells undergo replicative senescence [35]. Maintaining the structure of telomeres is key to the survival of cancer cells. Hence, 85–95% of human cancers express telomerase, while the other types of cancer cells maintain a sufficient length of telomeres through a mechanism that is based on DNA recombination called alternative lengthening of telomeres (ALT) [36,37]. Thus, it can be inferred that telomeres are essential for the survival of normal cells and cancer cells.

The regulation of gene expression is another function of telomeres that has recently attracted attention. Telomeres are a type of constitutive heterochromatin along with centromeres, and they form a huge complex that inhibits gene transcription by means of unique histone modifications [38,39,40]. Heterochromatin protein 1α (HP1α), which functions in telomere protection as well as the propagation of heterochromatic marks such as trimethylation at the ninth lysine of histone H3, is one such representative histone modifier [41,42].

**Figure 1:**
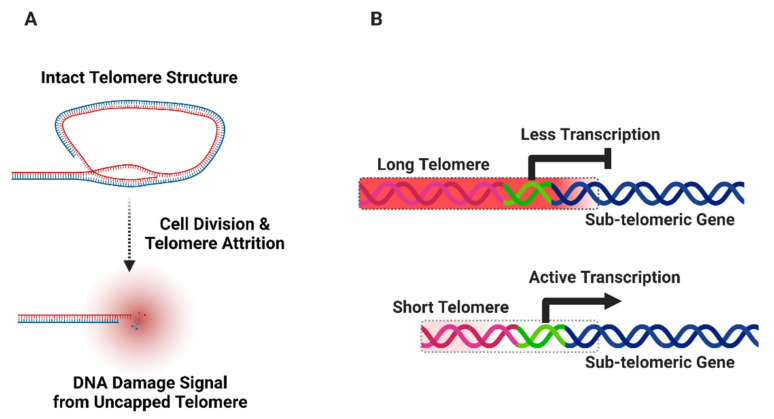
Roles of the telomeres in cells. (**A**) Genome integrity: Telomeres are key to maintaining the integrity of the genome. Telomeres of sufficient length form D-loop and T-loop structure, which prevent the generation of DNA damage signals and block telomere fusion. A decrease in telomere length causes cellular senescence by generating a “too short” or “uncapped” signal. (**B**) Telomere position effect: Another function of telomeres is to regulate the expression of nearby genes. Long telomeres inhibit transcription by suppressing the activity of promoters of nearby genes. This effect is gradually reduced due to the decrease in telomere length with the progression of replicative senescence. Created with BioRender.com (accessed on 28 October 2021).

The human genome has many sub-telomeric genes that are distributed within a range of tens to hundreds of kilobases from the loci of the telomeres. The expression of these genes can be regulated through the telomere position effect (TPE), a fact best exemplified by the *Saccharomyces cerevisiae* yeast model [43]. In 2001, it became known that the expression of sub-telomeric genes is regulated in a manner that is correlated to the length of telomeres in human cells [44], and this has gradually attracted the attention of many scientists. Figure 1 illustrates the main roles of telomeres as being the preservation of genome integrity and the regulation of gene expression.

Changes in gene expression of human cells due to aging and alterations in physiological functions are the main phenomena that are associated with aging [45]. Cellular aging and premature senescence can be explained in terms of the accumulated corruption of genetic information due to external causes such as genotoxic stress from ultraviolet rays, as well as due to internal stress such as spontaneous DNA repair/replication errors or the generation of reactive oxygen species (ROS) during mitochondrial respiration [46,47]. All of these accumulated alterations change the three-dimensional genome architecture, and the subsequent rearrangement of telomere heterochromatin affects specific gene expression, which is known as TPE. This review focuses on structural changes in the genome architecture via the loss of telomere repeats during replicative senescence, which cause significant changes in the mRNA expression of sub-telomeric genes as well as that of distal genes that are several megabases away from the telomeres.

## 3. Telomere Position Effect (TPE)

Telomeres, being constitutive heterochromatin domains, also perform the crucial function of strongly suppressing the expression of nearby subtelomeric genes. This is called the telomere position effect (TPE). The phenomenon was extensively studied in the yeast model at first [43], but it was observed that the TPE also occurs in flies, mice, and humans [44,48,49]. One of the significant outcomes of this finding was that the TPE could work as a key epigenetic mechanism for triggering the profound change in expression of various genes with aging. Differential expression of many genes in various human tissues by aging is a well-known phenomenon and is closely related to the development of certain diseases [50,51,52]. It is well known that the accumulation of mutations, destabilization of metabolic homeostasis, and the deregulation of protein synthesis collectively induce the aging phenotypes [52,53,54]. However, although the linear regression of telomere length is also another distinct hallmark during replicative senescence of human proliferative cells, few studies on the roles of TPE in gene regulation by aging have been conducted. Since most human cancers have very short telomeres [55,56], the expression of tumor-specific genes that are suppressed in young and long telomere cells may be regulated by the TPE mechanism. A recent study has also shown that long-ranged TPE can affect the expression of h*TERT* that are 1.2 mega bases away from chromosome 5*p* by forming telomere loops in CD4^+^ T lymphocytes and promyelocytic cancer cells [57]. Since telomerase expression is observed in most cancer cells and the monoallelic expression of the h*TERT* gene by promoter mutation is frequent in human cancers [58,59,60], further research on the relationship between the telomerase reactivation and TPE is necessary for the elucidation of the mechanism [61,62].

Another representative disease that is related to the altered gene regulation by telomere shortening includes FSHD (facioscapulohumeral muscular dystrophy), and the role of TPE during the late onset of the FSHD in aged patients has been well characterized [63]. Studies have documented the involvement of TPE in the abnormal expression of the *DUX4* gene, which could be a cause of FSHD with other transcription factors by regulating downstream target genes [64,65]. The expression of pathogenic *DUX4* is associated with epigenetic dysregulation of subtelomeric D4Z4 microsatellite at chromosome 4*q*35 [66]. Another recent study found that the expression of *SORBS2* (Sorbin and SH3 domain-containing protein 2) is also regulated by telomere length in the muscles of FSHD patients [67]. Therefore, accumulating evidence for the close relationship between the telomere length and regulation of gene expression suggest that the roles of telomere-dependent gene regulation is important in the development of specific diseases during replicative senescence of human cells. This review comprises of an overview of recent updates in the regulation of gene expression by telomeres and the prediction of future prospects for this area of research.

## 4. Telomere Position Effect-Over Long Distances (TPE-OLD)

Epigenetic regulatory mechanisms, such as histone modification, DNA modification, and chromatin remodeling are known to play crucial roles in complex mammalian gene regulatory networks [68,69]. Chromatin interaction dynamics by means of gene looping are epigenetic regulatory mechanisms that have been widely investigated recently [70,71,72]. Research pertaining to these mechanisms has provided answers for fundamental questions regarding developmental processes and pathogeneses like cancer and neurodegenerative diseases [73,74,75,76,77].

It also has been recently shown that telomeres, which are repetitive sequences at the ends of chromatin, make looping structures that are complementary to internal genomic regions that might be several mega bases away from the chromatin ends [57,78,79,80]. The role of this interaction between telomeres and particular genomic loci still needs to be elucidated, but there is growing evidence that this telomere looping makes the targeted locus silent. This is called the telomere position effect-over long distances (TPE-OLD) or telomere looping. Telomere looping suppresses gene expression in the target genomic region, but the detailed mechanisms are largely unknown. Table 1 shows a list of significant candidate genes whose mRNA expression was changed by telomere length via quantitation of mRNA transcripts. There is also a group of genes whose expression is decreased by telomere shortening. We consider this to be the result of an indirect effect by the action of the proteins whose expression is increased by TPE-OLD.

The loss of telomeres due to cell division causes gradual structural changes in the genome. The changes that are accumulated over time can completely change the position of each component of the genome that is related to gene expression because regulatory elements such as promoters, enhancers, silencers, and insulators could work in a three-dimensional way [86]. Since telomeres are a type of heterochromatin, the location of the telomeres in the genome is closely related to the regulation of target gene expression.

**Figure 2:**
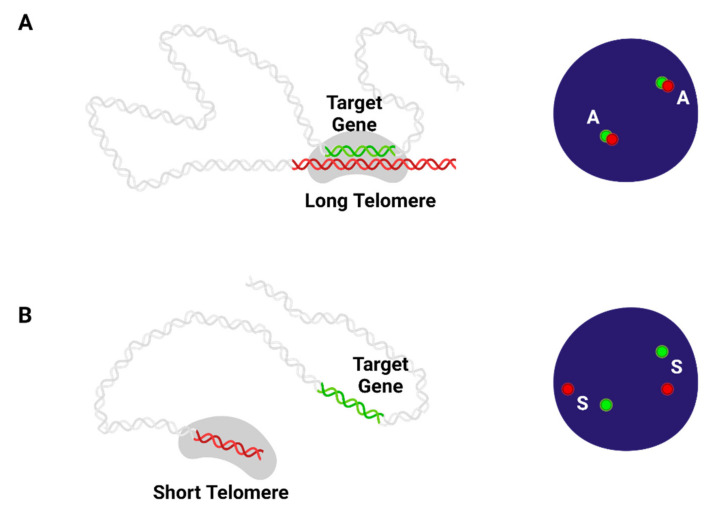
Telomere Position Effect- Over Long Distances (TPE-OLD): Since telomeres are heterochromatin, various trans-acting factors could be recruited by them for suppression of the genes at the target loci. The human genome, like proteins, has a complex three-dimensional folded structure. Thus, telomeres can not only affect nearby genes but also regulate distant genes that are several mega bases away. (**A**) Long telomere can make looping a structure against the target gene to suppress its expression. (**B**) Progressive telomere shortening disengages the telomere looping so that the mRNA expression of the target gene could be triggered. Created with BioRender.com (accessed on 28 October 2021).

The expression of specific genes is regulated by changes in telomere length because the three-dimensional folding structure of the genome changes through consecutive telomere attrition (Figure 2). The phenomenon called TPE-OLD or telomere looping thus refers to the event when telomeres that are of sufficient length form a looping structure that acts against a specific locus to inhibit the expression of a target gene [57,79]. Extensive studies on the factors mediating the TPE-OLD are yet to be performed. However, in a report that studied the association between h*TERT* and the chromosome 5*p* telomere, TRF2 promoted the interaction between the 5*p* telomere and internal telomeric sequences located near the h*TERT* locus [80]. Further, in the same study, the amounts of CTCF (CCCTC-binding factor) and LDB1 (LIM domain binding 1) proteins affected TPE-OLD so that the general chromatin looping mechanism would also have a significant effect on TPE-OLD.

Many cell divisions over time cause a critical shortening of telomere length, followed by an increase in the expression of certain genes by disengaging the looped complex between the telomere and target loci. However, the molecular and cell biological mechanisms underlying this phenomenon are still largely unknown.

Another important question may be whether TPE-OLD occurs only inside the same chromosome. All of the reports to date that suggest the physical association of telomeres with specific loci have only shown binding within the same chromosome [67,79,80].

Since eukaryotic chromosomes are confined to discrete regions (chromosome territories) during the interphase [87], this seems relevant. However, since the association between heterogeneous chromosomes is not impossible, further research on this topic is necessary to understand the mechanism of TPE-OLD.

This non-canonical function of telomeres is highly important in age-related diseases because telomere shortening disengages the looping interactions and this is followed by the activation of pathogenic genes [57,78]. Telomere shortening is one of the most prominent phenomena in aging cells and plays an important role in cell physiology before the onset of intensive DNA damage signals and genome aneuploidy [67]. Therefore, the epigenetic changes that are responsible for gene expression and splicing during telomere shortening should be elucidated to gain a better understanding of multiple pathogeneses during aging.

Short telomere length is associated with tumors, Alzheimer’s disease, and diabetes mellitus [55,87,88,89,90]. The cause-effect relationship is unclear and requires further studies, but the decreased telomere length and changes in gene expression resulting from TPE-OLD may be related to the pathogenesis of the diseases. A recent report has shown that human bronchial epithelial cells with over 400 population doubling with critically short telomeres exhibited low telomerase activity that could be considered one of the essential prerequisites for cancer transformation [91]. The study suggested that telomerase activity in cells with critically short telomeres is mandatory for clonal growth of epithelial cells. This was based on the observation that genome editing against telomerase reverse transcriptase (h*TERT*) or treatment with 6-thio-2′-deoxyguanosine (6-thio-dG) inhibited the formation of colonies.

Mechanisms related to TPE-OLD also have been reported recently based on a study of giant cell tumor of bone (GCTB), which is a rare bone tumor [92]. The authors suggested that the reactivation of h*TERT* transcription is related to the length of the telomeres, suggesting that TPE-OLD may be involved in the acquisition of telomerase reactivation during the development of GCTB. Another significant disease in which TPE-OLD is likely involved is triple-negative breast cancer (TNBC). Transcriptional coactivator with PDZ-binding motif (TAZ) is highly expressed in TNBC and is involved in the Hippo signaling system to regulate cell growth and death [93]. The reactivation of h*TERT* transcription was found only in late passages of TAZ knockdown cells with shortened telomeres, which indirectly suggests that TPE-OLD may be one of the major mechanisms in the development of certain types of breast cancer [94]. In previous studies, shelterin components that were not located in the telomeres were shown to be involved in the expression of specific genes [82,95], implying that the regulation of gene expression in certain genomic regions far from the telomeres might be influenced by TPE-OLD. Since the expression of telomerase in cancer cells is nearly universal [96], studying telomerase reactivation is essential for understanding the mechanism and treatment of cancer where TPE-OLD might play a major role in the epigenetic mechanisms involved therein.

The role of local genome structure change by telomere shortening at each chromosome end might explain why senescent cells show significant alterations in gene expression, followed by a decline in cell functions that are related to proliferation and survival. Research on the specific alterations in genome architecture in terms of long-range interaction between telomeres and particular loci during human aging will expand the understanding of the fundamental biology of geriatric diseases.

Considerable technical difficulties in simulating the complex and multifaceted processes of cellular aging hinder the study of the roles of the telomere position effect from being able to clarify the mechanisms that are involved in the onset of aging. In particular, an increase in population doubling during continuous cultivation of cells (in vitro aging) affects many components of aging factors so that the results could be significantly different from human aging in vivo [97,98]. Controlling the variables that are associated with aging and interpreting the results is difficult in the case of in vitro aging due to confounding effects. Utilizing aged tissues or aged animal models for the study of aging is also not fruitful because all of the biological changes that are observed might be derived from multiple causes which makes the results uninterpretable. Therefore, a model involving the generation of controlled senescent cells with the same genetic background and population doubling would be one of the best fits for the study of aging in relation to discovering candidate genes whose expression changes only in association with the length of telomeres [79]. With such models, the analyses would be carried out before the DNA damage response (DDR) occurs. Hence, these models will provide a convincing explanation of what happens in the cell at the early and middle stages of aging, This is unlike the many published aging studies that analyzed endpoint phenotypes of aging which could have resulted from multiple factors such as DNA damage, genomic instability, epigenetic alterations, loss of proteostasis, or mitochondrial dysfunction [45,99]. Studying the effect of telomere position, which is progressively influenced by changes in genome architecture due to telomere attrition, will facilitate the understanding and treatment certain geriatric diseases.

## 5. Conclusions

Conformational changes in chromatin, DNA modification, and histone modification are considered to be key factors in various human pathologies [100,101,102]. These epigenetic changes play an essential role in complex gene regulatory networks. Among them, chromatin interaction/conformation dynamics, such as gene looping, have been actively studied recently. Research on these mechanisms provides answers to some fundamental questions in the field of gene regulation [70,72,103]. In previous studies, the three-dimensional arrangement of genomes has been shown to have essential functions in the regulation of gene expression. An understanding of the temporal dynamics of gene looping and topology-associated domains (TADs) has addressed several fundamental questions regarding mammalian gene expression [104,105].

It has also been shown that telomere shortening causes local genomic structural changes, which is followed by global gene expression perturbation. The development of various cancers, such as colorectal and prostate cancers, is closely related with telomere shortening [21,106,107]. Therefore, there is a need for further understanding of telomere shortening and regulation of related genes for proper treatment of cancers.

For example, the h*TERT* promoter mutation occurs in various cancers and is known to play an important role in the survival of tumor cells through telomerase reactivation [60,108,109]. TPE-OLD between the h*TERT* locus and the 5*p* telomere may be related to mutation and hypomethylation of the h*TERT* promoter. A recent report suggests that hypomethylation of the mutated h*TERT* promoter might be cooperatively induced by disengaged telomere looping, which could cause significant differences in CpG island methylation in h*TERT*-promoter mutant cell lines [110]. Many studies have shown interest in the involvement of TPE-OLD in h*TERT* promoter mutation in the early development of cancer, thus making it necessary to conduct extensive research in this area [60,111,112,113]. In the previous TPE-OLD study on h*TERT*, 20–40% of the h*TERT* locus was still bound to the telomere in short telomere or aged fibroblasts [80] contrary to other TPE-OLD genes such as *C1S*, *ISG15*, and *SORBS2* [67,79]. This may be related to the characteristic monoallelic expression of h*TERT* through promoter mutation in cancer cells, unlike most genes that exhibit biallelic expression [59]. It may be possible to study whether telomere looping is specifically disengaged at the mutated promoter to examine if the telomere looping could be one of the mechanisms that suppresses the promoter mutation of h*TERT* in young and long telomere cells. This research will facilitate the understanding of the mechanisms of cancer development and telomerase reactivation, which is essential for developing therapeutic agents.

The phenomenon of telomere looping that results in the inactivation of target genes was recently discovered [57,80]. However, an understanding of the detailed mechanism that is involved in telomere looping requires further investigation. This function of telomeres is very important in the development of geriatric diseases because the decrease in telomere length with aging induces the expression of target pathogenic genes [78]. Although these mechanisms are important in various types of tissues, their roles have not been well studied. Existing theories and analysis methods do not fully explain the changes in the gene expression or splicing of specific genes. The decrease in the length of telomeres is one of the most notable aging-related changes in the cell, and this phenomenon occurs gradually before the onset of the DNA damage response that occurs in the early stages of aging [67]. Therefore, the change in telomere length and the subsequent regulation of the expression of various genes must be studied to understand various pathological phenomena that are related to aging. In addition, a study on the conformational change of the local genome architecture in relation to the change in telomere length is expected to provide insights into the issue of specific pathological genes being expressed only in senescent cells. The roles of various elements of the human genome cannot be interpreted with a linear model but must be interpreted based on a complex three-dimensional folded structure [86]. The decrease in the length of telomeres is the most representative causal factor for structural changes in the genome as aging progresses. Thus, epigenetic studies of changes in the genome structure due to aging will elucidate the mechanisms of geriatric diseases including cancer.

## Figures and Tables

**Table 1:** List of genes whose expression changes due to telomere length have been verified through quantitative PCR.

Gene	Genomic Location ^1^	Expression in Short Telomere	Tissue	Telomeric Interaction ^2^	Ref.
*ISG15*	1.0 Mb from 1*p*	Increase	BJ Skin Fibroblast Mammary Epithelial Cell Kidney Epithelial Cell	O	[57,81]
*DUX4*	50 Kb from 4*q*	Increase	Myoblast	O	[78]
*SORBS2*	4.4 Mb from 4*q*	Increase	Myoblast	O	[67]
*TERT*	1.2 Mb from 5*p*	Increase	BJ Skin Fibroblast	O	[80]
*NRN1*	6.0 Mb from 6*p*	Increase	BJ Skin Fibroblast	?	[79]
*DSP*	7.5 Mb from 6*p*	Increase	BJ Skin Fibroblast Myoblast	O	
*BMP6*	7.9Mb from 6*p*	Increase	BJ Skin Fibroblast Myoblast	?	
*C1S*	7.0 Mb from 12*p*	Increase	BJ Skin Fibroblast	O	
*FOXM1*	2.9 Mb from 12*p*	Increase	Myoblast	?	
*TSPAN9*	3.3 Mb from 12*p*	Increase			
*TIGAR*	4.3 Mb from 12*p*	Increase			
*CCND2*					
*AKAP3*	4.7Mb from 12*p*	Increase			
*GALNT8*					
*NDUFA9*					
*ANO2*	5.9 Mb from 12*p*	Increase			
*CD163L*	7.4 Mb from 12*p*	Increase			
*FOXC1*	1.6 Mb from 6*p*	Decrease	BJ Skin Fibroblast		
*TEAD4*	3.0 Mb from 12*p*	Decrease	Myoblast		
*CD9*	6.2 Mb from 12*p*				
*RPA2*	27.9 Mb from 1*p*	Decrease	HT1080 Fibrosarcoma	?	[82]
*INHA*	22.6 Mb from 2*q*	Decrease	HT1080 Fibrosarcoma MRC5 Lung Fibroblast		
*OBSL1*					
*PDGFRβ*	31.4 Mb from 5*q*	Increase	HT1080 Fibrosarcoma MRC5 Lung Fibroblast		
*WRNIP1*	2.8 Mb from 6*p*	Increase	HT1080 Fibrosarcoma MRC5 Lung Fibroblast		
*CDKN1A*	36.7 Mb from 6*p*	Decrease	HT1080 Fibrosarcoma MRC5 Lung Fibroblast		
*PSMC2*	56.0 Mb from 7*q*	Decrease	HT1080 Fibrosarcoma MRC5 Lung Fibroblast		
*BRSK2*	1.4 Mb from 11*p*	Decrease	HT1080 Fibrosarcoma MRC5 Lung Fibroblast		
*EEF1G*	72.5 Mb from 11*q*	Decrease	HT1080 Fibrosarcoma MRC5 Lung Fibroblast		
*ATN1*	6.9 Mb from 12*p*	Decrease	HT1080 Fibrosarcoma MRC5 Lung Fibroblast		
*TBC1D2B*	24.0 Mb from 15*q*	Decrease	HT1080 Fibrosarcoma MRC5 Lung Fibroblast		
*ANXA2*	41.7 Mb from 15*q*	Decrease	HT1080 Fibrosarcoma MRC5 Lung Fibroblast		
*SAMD14*	33.2 Mb from 17*q*	Decrease	HT1080 Fibrosarcoma MRC5 Lung Fibroblast		
*THRA*	43.2 Mb from 17*q*	Increase	HT1080 Fibrosarcoma		
*PSMA8*	54.2 Mb from 18*q*	Decrease	HT1080 Fibrosarcoma MRC5 Lung Fibroblast		
*RYR2*	11.9 Mb from 1*q*	Decrease	MRC5 Lung Fibroblast		
*KCNH2*	8.4 Mb from 7*q*	Decrease	MRC5 Lung Fibroblast		
*PDE3A*	20.7 Mb from 12*p*	Decrease	MRC5 Lung Fibroblast		
*THRA*	43.2 Mb from 17*q*	Decrease	MRC5 Lung Fibroblast		
*SMAD7*	31.5 Mb from 18*q*	Decrease	MRC5 Lung Fibroblast		
*EHMT1*	558 Kb from 9*q*	Increase	Abdominal Skin Fibroblast	?	[83]
*ZNF10*	116 Kb from 10*p*	Increase	Abdominal Skin Fibroblast		
*RASA3*	232 Kb from 10*p*	Increase	Abdominal Skin Fibroblast		
*ZMYND11*	254 Kb from 10*p*	Increase	Abdominal Skin Fibroblast		
*DIP2C*	470 Kb from 10*p*	Increase	HCA2 Skin Fibroblast	?	[84]
*CPNE7*	762 Kb from 13*q*	Increase	HCA2 Skin Fibroblast		
*GAS8*	193 Kb from 16*q*	Increase	HCA2 Skin Fibroblast		
*DBNDD1*	211 Kb from 16*q*	Increase	HCA2 Skin Fibroblast		
*CDK10*	652 Kb from 16*q*	Increase	HCA2 Skin Fibroblast		
*SCGC1C1*	32 Kb from 17*p*	Increase	HCA2 Skin Fibroblast		
*RBFA*	236 Kb from 18*q*	Increase	HCA2 Skin Fibroblast		
*FRG1*	172 Kb from 4*q*	Decrease	HCA2 Skin Fibroblast		
*TRIM7*	288 Kb from 5*q*	Decrease	HCA2 Skin Fibroblast		
*FOXD4*	127 Kb from 9*p*	Decrease	HCA2 Skin Fibroblast		
*CYP2E1*	175 Kb from 10*q*	Decrease	HCA2 Skin Fibroblast		
*IQSEC3*	158Kb from 12*p*	Decrease	HCA2 Skin Fibroblast		
*CHAMP1*	29 Kb from 13*q*	Decrease	HCA2 Skin Fibroblast		

^1^ All distances were estimated through the Genome Browser [85]. However, if there was information on distances in the original article, it was used as indicated. ^2^ The physical interaction between the indicated gene and telomeric region has been suggested by 3D-FISH (Fluorescence in situ hybridization).

## References

[1] Capper R., Britt-Compton B., Tankimanova M., Rowson J., Letsolo B., Man S., Haughton M., Baird D.M. (2007). The nature of telomere fusion and a definition of the critical telomere length in human cells. Genes Dev..

[2] Tanaka H., Abe S., Huda N., Tu L., Beam M.J., Grimes B., Gilley D. (2012). Telomere fusions in early human breast carcinoma. Proc. Natl. Acad. Sci. USA.

[3] Murnane J.P. (2010). Telomere Loss as a Mechanism for Chromosome Instability in Human Cancer. Cancer Res..

[4] Lototska L., Yue J., Li J., Giraud-Panis M., Songyang Z., Royle N.J., Liti G., Ye J., Gilson E., Mendez-Bermudez A. (2020). Human RAP 1 specifically protects telomeres of senescent cells from DNA damage. EMBO Rep..

[5] Van Steensel B., Smogorzewska A., de Lange T. (1998). TRF2 Protects Human Telomeres from End-to-End Fusions. Cell.

[6] Rai R., Chen Y., Lei M., Chang S. (2016). TRF2-RAP1 is required to protect telomeres from engaging in homologous recombination-mediated deletions and fusions. Nat. Commun..

[7] Mao P., Liu J., Zhang Z., Zhang H., Liu H., Gao S., Rong Y.S., Zhao Y. (2016). Homologous recombination-dependent repair of telomeric DSBs in proliferating human cells. Nat. Commun..

[8] Janoušková E., Nečasová I., Pavloušková J., Zimmermann M., Hluchý M., Marini V., Nováková M., Hofr C. (2015). Human Rap1 modulates TRF2 attraction to telomeric DNA. Nucleic Acids Res..

[9] Moyzis R.K., Buckingham J.M., Cram L.S., Dani M., Deaven L.L., Jones M.D., Meyne J., Ratliff R.L., Wu J.R. (1988). A highly conserved repetitive DNA sequence, (TTAGGG)n, present at the telomeres of human chromosomes. Proc. Natl. Acad. Sci. USA.

[10] Allsopp R.C., Vaziri H., Patterson C., Goldstein S., Younglai E.V., Futcher A.B., Greider C.W., Harley C.B. (1992). Telomere length predicts replicative capacity of human fibroblasts. Proc. Natl. Acad. Sci. USA.

[11] Chen W., Kimura M., Kim S., Cao X., Srinivasan S.R., Berenson G.S., Kark J.D., Aviv A. (2011). Longitudinal versus Cross-sectional Evaluations of Leukocyte Telomere Length Dynamics: Age-Dependent Telomere Shortening is the Rule. J. Gerontol. Ser. A Boil. Sci. Med. Sci..

[12] Werner B., Beier F., Hummel S., Balabanov S., Lassay L., Orlikowsky T., Dingli D., Brümmendorf T.H., Traulsen A. (2015). Reconstructing the in vivo dynamics of hematopoietic stem cells from telomere length distributions. eLife.

[13] Lin Y., Damjanovic A., Metter E.J., Nguyen H., Truong T., Najarro K., Morris C., Longo D.L., Zhan M., Ferrucci L. (2015). Age-associated telomere attrition of lymphocytes in vivo is co-ordinated with changes in telomerase activity, composition of lymphocyte subsets and health conditions. Clin. Sci..

[14] Nordfjäll K., Svenson U., Norrback K.-F., Adolfsson R., Lenner P., Roos G. (2009). The Individual Blood Cell Telomere Attrition Rate Is Telomere Length Dependent. PLoS Genet..

[15] O’Sullivan R.J., Karlseder J. (2010). Telomeres: Protecting chromosomes against genome instability. Nat. Rev. Mol. Cell Biol..

[16] Greider C. (1999). Telomeres Do D-Loop–T-Loop. Cell.

[17] A Blasco M. (2007). Telomere length, stem cells and aging. Nat. Chem. Biol..

[18] Griffith J.D., Comeau L., Rosenfield S., Stansel R.M., Bianchi A., Moss H., de Lange T. (1999). Mammalian Telomeres End in a Large Duplex Loop. Cell.

[19] Sarek G., Kotsantis P., Ruis P., Van Ly D., Margalef P., Borel V., Zheng X.-F., Flynn H., Snijders B., Chowdhury D. (2019). CDK phosphorylation of TRF2 controls t-loop dynamics during the cell cycle. Nature.

[20] Tomaska L., Nosek J., Kar A., Willcox S., Griffith J.D. (2019). A New View of the T-Loop Junction: Implications for Self-Primed Telomere Extension, Expansion of Disease-Related Nucleotide Repeat Blocks, and Telomere Evolution. Front. Genet..

[21] Neumann A.A., Reddel R. (2002). Telomere Maintenance and Cancer? Look, No Telomerase. Nat. Rev. Cancer.

[22] Wang F., Lei M. (2011). Human Telomere POT1-TPP1 Complex and Its Role in Telomerase Activity Regulation. Methods Mol. Biol..

[23] De Lange T. (2005). Shelterin: The protein complex that shapes and safeguards human telomeres. Genes Dev..

[24] Lim C.J., Zaug A.J., Kim H.J., Cech T.R. (2017). Reconstitution of human shelterin complexes reveals unexpected stoichiometry and dual pathways to enhance telomerase processivity. Nat. Commun..

[25] Diotti R., Loayza D. (2011). Shelterin complex and associated factors at human telomeres. Nucleus.

[26] Kibe T., Zimmermann M., de Lange T. (2016). TPP1 Blocks an ATR-Mediated Resection Mechanism at Telomeres. Mol. Cell.

[27] Hockemeyer D., Collins K. (2015). Control of telomerase action at human telomeres. Nat. Struct. Mol. Biol..

[28] Shayand J.W., Wright W.E. (2005). Senescence and immortalization: Role of telomeres and telomerase. Carcinogenesis.

[29] Shay J.W., Wright W.E., Werbin H. (1991). Defining the molecular mechanisms of human cell immortalization. Biochim. Biophys. Acta Bioenerg..

[30] Michie C. (2001). Do telomeres help define the genes expressed during ageing?. Trends Mol. Med..

[31] Di Micco R., Fumagalli M., Cicalese A., Piccinin S., Gasparini P., Luise C., Schurra C., Garre’ M., Nuciforo P., Bensimon A. (2006). Oncogene-induced senescence is a DNA damage response triggered by DNA hyper-replication. Nature.

[32] Poele R.H., Okorokov A.L., Jardine L., Cummings J., Joel S.P. (2002). DNA Damage Is Able to Induce Senescence in Tumor Cells In Vitro and In Vivo. Cancer Res..

[33] Shay J.W., Wright W. (2019). Telomeres and telomerase: Three decades of progress. Nat. Rev. Genet..

[34] A Ulaner G. (1997). Developmental regulation of telomerase activity in human fetal tissues during gestation. Mol. Hum. Reprod..

[35] Collins K., Mitchell J.R. (2002). Telomerase in the human organism. Oncogene.

[36] Trybek T., Kowalik A., Góźdź S., Kowalska A. (2020). Telomeres and telomerase in oncogenesis (Review). Oncol. Lett..

[37] Cesare A., Reddel R. (2010). Alternative lengthening of telomeres: Models, mechanisms and implications. Nat. Rev. Genet..

[38] Schoeftner S., A Blasco M. (2009). A ‘higher order’ of telomere regulation: Telomere heterochromatin and telomeric RNAs. EMBO J..

[39] Wang J., Cohen A.L., Letian A., Tadeo X., Moresco J.J., Liu J., Yates J.R., Qiao F., Jia S. (2016). The proper connection between shelterin components is required for telomeric heterochromatin assembly. Genes Dev..

[40] Cubiles M.D., Barroso S., Vaquero-Sedas I.M., Enguix A., Aguilera A., Vega-Palas A.M. (2018). Epigenetic features of human telomeres. Nucleic Acids Res..

[41] Chow T.T., Shi X., Wei J.-H., Guan J., Stadler G., Huang B., Blackburn E.H. (2018). Local enrichment of HP1alpha at telomeres alters their structure and regulation of telomere protection. Nat. Commun..

[42] Roach R.J., Garavís M., González C., Jameson G.B., Filichev V., Hale T.K. (2020). Heterochromatin protein 1α interacts with parallel RNA and DNA G-quadruplexes. Nucleic Acids Res..

[43] Tham W.-H., Zakian A.V. (2002). Transcriptional silencing at Saccharomyces telomeres: Implications for other organisms. Oncogene.

[44] Baur J.A., Zou Y., Shay J.W., Wright W.E. (2001). Telomere Position Effect in Human Cells. Science.

[45] DiLoreto R., Murphy C.T. (2015). The cell biology of aging. Mol. Biol. Cell.

[46] Fan D.N.Y., Schmitt C.A. (2019). Genotoxic Stress-Induced Senescence. Methods Mol. Biol..

[47] Maynard S., Fang E.F., Scheibye-Knudsen M., Croteau D.L., Bohr V.A. (2015). DNA Damage, DNA Repair, Aging, and Neurodegeneration. Cold Spring Harb. Perspect. Med..

[48] Weuts A., Voet T., Verbeeck J., Lambrechts N., Wirix E., Schoonjans L., Danloy S., Marynen P., Froyen G. (2012). Telomere length homeostasis and telomere position effect on a linear human artificial chromosome are dictated by the genetic background. Nucleic Acids Res..

[49] Mason J.M., Konev A.Y., Biessmann H. (2003). Telomeric position effect in drosophila melanogaster reflects a telomere length control mechanism. Genetica.

[50] Glass D., Viñuela A., Davies M.N., Ramasamy A., Parts L., Knowles D., Brown A.A., Hedman Å.K., Small K.S., Buil A. (2013). Gene expression changes with age in skin, adipose tissue, blood and brain. Genome Biol..

[51] Yang J., Huang T., Petralia F., Long Q., Zhang B., Argmann A.C., Zhao Y., Mobbs C., Schadt E.E., The GTEx Consortium (2015). Synchronized age-related gene expression changes across multiple tissues in human and the link to complex diseases. Sci. Rep..

[52] Frenk S., Houseley J. (2018). Gene expression hallmarks of cellular ageing. Biogerontology.

[53] Brink T.C., Demetrius L., Lehrach H., Adjaye J. (2008). Age-related transcriptional changes in gene expression in different organs of mice support the metabolic stability theory of aging. Biogerontology.

[54] Anisimova A.S., Meerson M.B., Gerashchenko M.V., Kulakovskiy I.V., Dmitriev S.E., Gladyshev V.N. (2020). Multifaceted deregulation of gene expression and protein synthesis with age. Proc. Natl. Acad. Sci. USA.

[55] Okamoto K., Seimiya H. (2019). Revisiting Telomere Shortening in Cancer. Cells.

[56] Shay J.W. (2013). Are Short Telomeres Predictive of Advanced Cancer?. Cancer Discov..

[57] Kim W., Shay J.W. (2018). Long-range telomere regulation of gene expression: Telomere looping and telomere position effect over long distances (TPE-OLD). Differentiation.

[58] Huang F.W., Bielski C.M., Rinne M.L., Hahn W.C., Sellers W.R., Stegmeier F., A Garraway L., Kryukov G. (2015). TERT promoter mutations and monoallelic activation of TERT in cancer. Oncogenesis.

[59] Stern J.L., Theodorescu D., Vogelstein B., Papadopoulos N., Cech T.R. (2015). Mutation of the TERT promoter, switch to active chromatin, and monoallelic TERT expression in multiple cancers. Genes Dev..

[60] Vinagre J., Almeida A., Pópulo H., Batista R., Lyra J., Pinto V., Coelho R., Celestino R., Prazeres H., Lima L. (2013). Frequency of TERT promoter mutations in human cancers. Nat. Commun..

[61] Min J., Shay J.W. (2016). TERT Promoter Mutations Enhance Telomerase Activation by Long-Range Chromatin Interactions. Cancer Discov..

[62] Akıncılar S.C., Khattar E., Boon P.L.S., Unal B., Fullwood M., Tergaonkar V. (2016). Long-Range Chromatin Interactions Drive Mutant TERT Promoter Activation. Cancer Discov..

[63] Sidlauskaite E., Le Gall L., Mariot V., Dumonceaux J. (2020). *DUX4* Expression in FSHD Muscles: Focus on Its mRNA Regulation. J. Pers. Med..

[64] Karpukhina A., Tiukacheva E., Dib C., Vassetzky Y.S. (2021). Control of DUX4 Expression in Facioscapulohumeral Muscular Dystrophy and Cancer. Trends Mol. Med..

[65] Banerji C.R.S., Zammit P.S. (2021). Pathomechanisms and biomarkers in facioscapulohumeral muscular dystrophy: Roles of DUX4 and PAX7. EMBO Mol. Med..

[66] Lemmers R.J.L.F., van der Maarel S., Van Deutekom J.C.T., Van Der Wielen M.J.R., Deidda G., Dauwerse H.G., Hewitt J., Hofker M., Bakker E., Padberg G.W. (1998). Inter- and intrachromosomal sub-telomeric rearrangements on 4q35: Implications for facioscapulohumeral muscular dystrophy (FSHD) aetiology and diagnosis. Hum. Mol. Genet..

[67] Robin J.D., Ludlow A.T., Batten K., Gaillard M.-C., Stadler G., Magdinier F., Wright W.E., Shay J.W. (2015). SORBS2 transcription is activated by telomere position effect–over long distance upon telomere shortening in muscle cells from patients with facioscapulohumeral dystrophy. Genome Res..

[68] Jaenisch R., Bird A. (2003). Epigenetic Regulation of Gene Expression: How the Genome Integrates Intrinsic and Environmental Signals. Nat. Genet..

[69] Kan R.L., Chen J., Sallam T. (2021). Crosstalk between epitranscriptomic and epigenetic mechanisms in gene regulation. Trends Genet..

[70] Tan-Wong S.M., Wijayatilake H.D., Proudfoot N.J. (2009). Gene loops function to maintain transcriptional memory through interaction with the nuclear pore complex. Genes Dev..

[71] Moabbi A.M., Agarwal N., El Kaderi B., Ansari A. (2012). Role for gene looping in intron-mediated enhancement of transcription. Proc. Natl. Acad. Sci. USA.

[72] Holwerda S., de Laat W. (2012). Chromatin loops, gene positioning, and gene expression. Front. Genet..

[73] Dekker J., Belmont A.S., Guttman M., Leshyk V.O., Lis J.T., Lomvardas S., Mirny L.A., O’Shea C.C., Park P.J., Ren B. (2017). The 4D nucleome project. Nature.

[74] Greenwald W.W., Li H., Benaglio P., Jakubosky D., Matsui H., Schmitt A., Selvaraj S., D’Antonio M., D’Antonio-Chronowska A., Smith E.N. (2019). Subtle changes in chromatin loop contact propensity are associated with differential gene regulation and expression. Nat. Commun..

[75] Behrends M., Engmann O. (2021). Loop Interrupted: Dysfunctional Chromatin Relations in Neurological Diseases. Front. Genet..

[76] Roix J.J., McQueen P.G., Munson P.J., A Parada L., Misteli T. (2003). Spatial proximity of translocation-prone gene loci in human lymphomas. Nat. Genet..

[77] Maurano M.T., Humbert R., Rynes E., Thurman R.E., Haugen E., Wang H., Reynolds A.P., Sandstrom R., Qu H., Brody J. (2012). Systematic Localization of Common Disease-Associated Variation in Regulatory DNA. Science.

[78] Stadler G., Rahimov F., King O.D., Chen J.C.J., Robin J., Wagner K.R., Shay J.W., Emerson C.P., Wright W.E. (2013). Telomere position effect regulates DUX4 in human facioscapulohumeral muscular dystrophy. Nat. Struct. Mol. Biol..

[79] Robin J.D., Ludlow A.T., Batten K., Magdinier F., Stadler G., Wagner K.R., Shay J.W., Wright W.E. (2014). Telomere position effect: Regulation of gene expression with progressive telomere shortening over long distances. Genes Dev..

[80] Kim W., Ludlow A.T., Min J., Robin J., Stadler G., Mender I., Lai T.-P., Zhang N., Wright W., Shay J.W. (2016). Regulation of the Human Telomerase Gene TERT by Telomere Position Effect-Over Long Distances (TPE-OLD): Implications for Aging and Cancer. PLoS Biol..

[81] Lou Z., Wei J., Riethman H., Baur J., Voglauer R., Shay J.W., Wright W.E. (2009). Telomere length regulates ISG15 expression in human cells. Aging.

[82] Mukherjee A.K., Sharma S., Sengupta S., Saha D., Kumar P., Hussain T., Srivastava V., Roy S.D., Shay J.W., Chowdhury S. (2018). Telomere length-dependent transcription and epigenetic modifications in promoters remote from telomere ends. PLoS Genet..

[83] Hernandez-Caballero E., Herrera-Gonzalez N., Salamanca-Gomez F., Arenas-Aranda D. (2009). Role of telomere length in subtelomeric gene expression and its possible relation to cellular senescence. BMB Rep..

[84] Ning Y., Xu J.-F., Li Y., Chavez L., Riethman H.C., Lansdorp P.M., Weng N.-P. (2003). Telomere length and the expression of natural telomeric genes in human fibroblasts. Hum. Mol. Genet..

[85] Kent W.J., Sugnet C.W., Furey T.S., Roskin K.M., Pringle T.H., Zahler A.M., Haussler A.D. (2002). The Human Genome Browser at UCSC. Genome Res..

[86] Herold M., Bartkuhn M., Renkawitz R. (2012). CTCF: Insights into insulator function during development. Development.

[87] Meaburn K., Misteli T. (2007). Chromosome territories. Nature.

[88] Zhu X., Han W., Xue W., Zou Y., Xie C., Du J., Jin G. (2016). The association between telomere length and cancer risk in population studies. Sci. Rep..

[89] Wang J., Dong X., Cao L., Sun Y., Qiu Y., Zhang Y., Cao R., Covasa M., Zhong L. (2016). Association between telomere length and diabetes mellitus: A meta-analysis. J. Int. Med Res..

[90] Forero D.A., González-Giraldo Y., López-Quintero C., Castro-Vega L.J., Barreto G.E., Perry G. (2016). Meta-analysis of Telomere Length in Alzheimer’s Disease. J. Gerontol. Ser. A Boil. Sci. Med. Sci..

[91] Peters-Hall J.R., Min J., Tedone E., Sho S., Siteni S., Mender I., Shay J.W. (2020). Proliferation of adult human bronchial epithelial cells without a telomere maintenance mechanism for over 200 population doublings. FASEB J..

[92] Forsyth R.G., Krenács T., Athanasou N., Hogendoorn P.C.W. (2021). Cell Biology of Giant Cell Tumour of Bone: Crosstalk between m/wt Nucleosome H3.3, Telomeres and Osteoclastogenesis. Cancers.

[93] Kedan A., Verma N., Saroha A., Shaked M., Müller A.-K., Nair N.U., Lev S. (2018). PYK2 negatively regulates the Hippo pathway in TNBC by stabilizing TAZ protein. Cell Death Dis..

[94] Yang L., Wang B., Jiao X., Zhou C., Chen S., Gao X., Sun W., Song S., Li J., Liu J. (2021). TAZ maintains telomere length in TNBC cells by mediating Rad51C expression. Breast Cancer Res..

[95] Teo H., Ghosh S., Luesch H., Ghosh A., Wong E.T., Malik N., Orth A., De Jesus P., Perry A., Oliver J.D. (2010). Telomere-independent Rap1 is an IKK adaptor and regulates NF-κB-dependent gene expression. Nat. Cell Biol..

[96] Roake C.M., Artandi S.E. (2020). Regulation of human telomerase in homeostasis and disease. Nat. Rev. Mol. Cell Biol..

[97] Schneider E.L., Mitsui Y. (1976). The relationship between in vitro cellular aging and in vivo human age. Proc. Natl. Acad. Sci. USA.

[98] Rubin H. (1997). Cell aging in vivo and in vitro. Mech. Ageing Dev..

[99] López-Otín C., Blasco M.A., Partridge L., Serrano M., Kroemer G. (2013). The Hallmarks of Aging. Cell.

[100] Huang C., A Sloan E., Boerkoel C.F. (2003). Chromatin remodeling and human disease. Curr. Opin. Genet. Dev..

[101] Boltsis I., Grosveld F., Giraud G., Kolovos P. (2021). Chromatin Conformation in Development and Disease. Front. Cell Dev. Biol..

[102] Kouzarides T. (2007). Chromatin Modifications and Their Function. Cell.

[103] Zhao B., Xi Y., Kim J., Sung S. (2021). Chromatin architectural proteins regulate flowering time by precluding gene looping. Sci. Adv..

[104] Cavalli G., Misteli T. (2013). Functional implications of genome topology. Nat. Struct. Mol. Biol..

[105] Mishra A., Hawkins R.D. (2017). Three-dimensional genome architecture and emerging technologies: Looping in disease. Genome Med..

[106] Rampazzo E., Bertorelle R., Serra L., Terrin L., Candiotto C., Pucciarelli S., Del Bianco P., Nitti D., De Rossi A. (2010). Relationship between telomere shortening, genetic instability, and site of tumour origin in colorectal cancers. Br. J. Cancer.

[107] Maser R.S., DePinho R.A. (2002). Connecting Chromosomes, Crisis, and Cancer. Science.

[108] Schwaederle M., Krishnamurthy N., Daniels G.A., Piccioni D.E., Kesari S., Fanta P.T., Schwab R.B., Patel S.P., Parker B.A., Kurzrock R. (2018). Telomerase reverse transcriptase promoter alterations across cancer types as detected by next-generation sequencing: A clinical and molecular analysis of 423 patients. Cancer.

[109] Heidenreich B., Kumar R. (2017). TERT promoter mutations in telomere biology. Mutat. Res. Mutat. Res..

[110] Hu K., Ghandi M., Huang F.W. (2021). Integrated evaluation of telomerase activation and telomere maintenance across cancer cell lines. eLife.

[111] Yuan X., Liu T., Xu D. (2020). Telomerase reverse transcriptase promoter mutations in thyroid carcinomas: Implications in precision oncology—A narrative review. Ann. Transl. Med..

[112] Yuan X., Dai M., Xu D. (2020). TERT promoter mutations and GABP transcription factors in carcinogenesis: More foes than friends. Cancer Lett..

[113] Guterres A.N., Villanueva J. (2020). Targeting telomerase for cancer therapy. Oncogene.

